# Characteristics of a thyroid carcinoma cell line derived from spinal metastasis

**DOI:** 10.1042/BSR20160403

**Published:** 2016-12-09

**Authors:** Zhenhua Zhou, Yan Li, Xu Yan, Xudong Wang, Su Chen, Jianru Xiao

**Affiliations:** * Department of Orthopaedic Oncology, Changzheng Hospital, The Second Military Medical University, Shanghai 200003, China; † Cancer Institute, Fudan University Shanghai Cancer Center, Department of Oncology, Shanghai Medical College, Fudan University, Shanghai 200032, China; ‡ Department of Orthopedics, The 455th Hospital of PLA, Shanghai 200052, China

**Keywords:** biomarker, cell line, metastatic thyroid carcinoma, spine, tumour biology

## Abstract

A thyroid carcinoma cell line named THY28 was established through primary culture of the surgical specimens, which were derived from a Chinese patient with spinal metastasis. The cell morphology, growth kinetics, cell cycle, chromosome number, cell capability of migration, tumorigenicity and cytogenetic features of the cell line were investigated. THY28 cells were subcultured *in vitro* for more than 50 passages with a human karyotype. The modal number of its chromosomes was mainly from 67 to 85. The doubling time of THY28 cells was 56 hours. The histopathological features of xenograft induced by THY28 cells were consistent with the characteristics of thyroid cancer. The biological and molecular properties of THY28 cells were not entirely consistent with those of other thyroid carcinoma cells such as SW579 and TT cells, indicating biological differences between primary and metastatic thyroid carcinoma cell lines. We have established a novel thyroid carcinoma cell line derived from spinal metastasis, which will provide a useful model for biological or therapeutic studies of thyroid carcinoma metastasis.

## Abstract (precis)

We established and characterized a new thyroid cancer cell line came from patients who presented spine metastasis; these cell lines could thus be new *in vivo* models for studying the biology including metastasis of thyroid cancer.

## INTRODUCTION

Metastasis of thyroid carcinoma has occurred in approximately 7% to 23% of patients at the time of diagnosis [1–3], which are usually associated with poor prognosis [4,5]. Metastasis of thyroid carcinoma is most common in lungs followed by skeletal system. Metastatic thyroid carcinoma spread to the spinal column can cause a number of sequelae including severe pain, instability and neurologic deficit, which is an intractable problem in patients with thyroid carcinoma. If left untreated, progressive myelopathy results in the loss of motor, sensory and autonomic functions [5–11]. For patients with spinal metastasis of thyroid carcinoma, the surgery is palliative, which is just to relieve symptoms of nerve compression and improve patients’ quality of life at the end-stage. Understanding the biological mechanism of metastasis in thyroid carcinoma is important for development of new treatment strategies aimed at metastasis.

For a complete investigation of the underlying mechanisms of metastasis of thyroid carcinoma, there is an absolute requirement to establish *in vitro* model systems to study the complex multistep process of thyroid carcinogenesis and metastasis at various stages of the disease. As we know, some cell lines have been established from tissues of primary thyroid carcinoma [12–16]. However, biological characteristics of cells derived from primary cancer were inevitably much different from those of metastases. Therefore, cell lines isolated from a metastatic cancer may be more appropriate for the study of molecular mechanisms involved in thyroid metastasis. In the present study, we established a novel thyroid papillary carcinoma cell line derived from spinal metastasis. We preliminarily investigated biological characteristics of this cell line and our findings could provide an *in vitro* model system to investigate metastatic events in thyroid carcinoma.

## MATERIALS AND METHODS

### Ethical approval

All procedures performed in studies involving animals were in accordance with the ethical standards of The United Kingdom Coordinating Committee on Cancer Prevention Research's Guidelines for the Welfare of Animals in Experimental Neoplasia. All experimental operations on animals were approved by Animal Ethics Committees of the Second Military Medical University. All procedures performed in studies involving human participants were in accordance with the ethical standards of the Clinical Research Ethics Committee of Second Military Medical University and with the 1964 Helsinki declaration and its later amendments or comparable ethical standards. Tumour tissues were obtained with informed consent, and the present study was approved by the Clinical Research Ethics Committee of the Second Military Medical University.

### Cell culture

Sterile sample of metastasis was obtained from a 60-year-old male Chinese patient diagnosed with metastatic thyroid papillary carcinoma in spine. The minced tumour tissues were placed in a tube supplemented with collagenase II (10 μl) in a thermostatic shaker at 37°C, 200 rpm, for 2 hours. Then tissues were filtered and the suspension was density gradient centrifuged for 30 minutes at 2000 g, and the middle cellular layer of suspension was carefully aspirated to move into culture dishes, supplemented with Dulbecco's modified Eagle's medium (DMEM) containing 10% FBS, 100 units/ml penicillin and 100 mg/ml streptomycin. Cells were placed in 25 cm^2^ culture flasks and kept at 37°C in a humidified atmosphere with 5% CO_2_. SW579 and TT cell lines (A.T.C.C.) were cultured in the same conditions as experimental controls.

### Analysis of cell cycle

THY28, SW579 and TT cells in the exponential growth phase were collected at a density of 1×10^6^ cells/ml. Cells were resuspended with 300 μl of PBS and added to precooled ethanol for fixation at 4°C overnight. Cells were centrifuged at 1000 g for 5 minutes and resuspended in 500 μl of PBS supplied with 100 units/ml RNaseA, then incubated at 37°C for 30 minutes. Propidium iodide (PI) was added to the cells to a final concentration of 50 μg/ml, and cells were incubated in the dark for 30 minutes. Cell numbers in each stage of cell cycle were counted by flow cytometry (BD Biosciences).

### Cell proliferation assay

Cell counting kit 8 (CCK8) (Dojindo Laboratories) was used in this assay as follows: THY28, SW579 and TT cells in the exponential growth phase were plated in 96-well plates at a density of 5000 cells per 200 μl and each plate had five controls. Cells were incubated for 5 days. Twenty microlitres of CCK8 were added to each well and co-incubated for 3.5 hours on day 1, 2, 4 and 5 respectively. Automatic microplate reader (BioTek) was used to measure the absorbance value in each well at 450 nm and the mean value was used to draw the cell growth curve. The independent cell proliferation assay was repeated three times.

### EM

Cells were centrifuged at 1000 g for 5 minutes and the sediment was washed with PBS, then fixed in 3% glutaraldehyde, postfixed in 1% osmium tetroxide, dehydrated through ascending series of ethanol and embedded in Epon. Semi-thin sections were cut and stained with 1% toluidine blue, and were used to select representative cell nests for light microscopic orientation. Ultra-thin sections were cut from each selected cell nest, stained with uranyl acetate and lead citrate and observed under a Hitachi 500 electron microscope.

### RNA extraction and quantitative real-time PCR

Cells were washed with precooled sterile PBS and lysed with TRIzol reagent (Invitrogen) according to the manufacturer's instructions. The concentrations of RNA were measured by a NanoDrop2000 (Thermo Fisher Scientific). cDNA was synthesized with the PrimeScript RT reagent kit (TaKaRa Bio) using 500 ng total RNA as a template. Quantitative real-time PCR (qRT-PCR) was performed using SYBR Premix Ex Taq (TaKaRa Bio) and  β-actin as an internal control. qRT-PCR was carried out using an ABI 7900HT instrument (Applied Biosystems). The primers used for qRT-PCR are listed in Table 1.

**Table 1:** Primers sequences for real time PCR

Genes	Forward primer(5′–3′)	Reverse primer(5′–3′)
*CK19*	GAAGGATGCTGAAGCCTGGT	CTGGGCTTCAATACCGCTGA
*CD15*	TGGCATGTAGGAAGCACCTG	GCACGTGGAACTAGGAGGTC
*TTF1*	GCGCTTTCGGAGGGTTAGA	GTGGCCCTGTCCTTGATGTT
*S100A4*	TGGTTTGGTGCTTCTGAGATGT	TGGAAGTCCACCTCGTTGTC
*NM23*	AAGCAGCTGGAAGGAACCAT	TTCACCACATTCAGCCCCTC
*CD117*	AAATCCATCCCCACACCCTG	AACCTTCCCGAAAGCTCCAG
*MMP9*	TCTATGGTCCTCGCCCTGAA	CATCGTCCACCGGACTCAAA
*CK20*	GGTCGCGACTACAGTGCATA	GAAGTCCTCAGCAGCCAGTT
*MCM3*	TGGAGTCATCCTGGGAACCT	TCAGCTCCCGAACTTTGCTC
*KI67*	CGTCCCAGTGGAAGAGTTGT	CGACCCCGCTCCTTTTGATA
*CD44*	TTACAGCCTCAGCAGAGCAC	TGACCTAAGACGGAGGGAGG
*Vimentin*	AGCCCGCTGAGACTTGAATC	CCTCTGTCCATCGACTTGCC

### Karyotype analysis

Colchicine (1×10^−5^ mol/l; Sigma–Aldrich) was added to the medium when cells were in the exponential growth phase. After being treated for 6 h, cells were collected and suspended in a hypotonic solution (0.04 mol/l KCl, 0.025 mol/l NaCl) followed by incubation at 37°C for 20 minutes. One ml of acetic acid and methanol (1:1) were added for fixation for 20 minutes. Then, cells were collected after centrifugation for 10 minutes and 0.2 ml acetic acid and methanol were added for cell dispersion. A drop of cell suspension was taken into the precooled glass slide and dispersed by the rubber suction bulb. Cells were observed under the microscope after Giemsa staining.

### Immunohistochemistry

Sections were cut and floated on to glass slides for immunohistochemistry staining. A standard technique as described recently [17] was used for immunohistochemistry. Antibodies to CK20, TTF1, MMP9, CD15, MCM3 and CD44 were obtained from Santa Cruz Biotechnology.

### *In vitro* cell migration and invasion assay

THY28 cells (1×10^5^) were added to the upper layer of the Transwell inserts (BD Biosciences). For the migration assay, cells were seeded in the upper chamber of each Transwell insert. For the invasion assay, the membrane was coated with Matrigel and cells were placed in the upper chamber. Then, the Transwell was transferred to 24-well plates filled with 0.6 ml complete medium and incubated at 37°C in an incubator containing 5% carbon dioxide for 24 hours. Transwell was taken out and the cells on the upper surface of the Transwell membrane were removed by wiping with cotton swabs. The cells on the lower surface of the Transwell membrane were fixed with 70% ethanol and stained with Crystal Violet. SW579 and TT cells were used as controls. The migration and invasion assay was performed in three independent wells, and four non-repetitive fields in each membrane were selected to count cells that migrated to the undersurface of the membrane under the optical microscope (Olympus).

### Flow cytometric analysis

THY28 cells (1×10^5^) were washed with PBS and harvested with trypsin and EDTA. Cell pellets were resuspended and incubated for 30 minutes at room temperature with 100-fold dilution of anti-CD15-PE, anti-CD117-PE and anti-CD44-FITC (BD PharMingen). Dead and damaged cells were eliminated with 7-AAD (BD Biosciences Pharmingen). Isotype controls (BD Biosciences) were used for control experiments. Dead cells were detected by PI staining. The results were analysed using software FlowJo version 7.2.4 software (Treestar).

### Tumorigenicity in immunodeficient mice

Ten 4-week-old BALB/cnu/nu mice were used to investigate the tumorigenic capacity of THY28 cells. Approximately 1×10^6^ THY28 cells resuspended in 200  μl of DMEM/Matrigel mixture (1:1) were injected subcutaneously into the right flank of mice. Tumour-bearing mice were killed 6 weeks after inoculation. Xenografts were excised and subjected to haematoxylin and eosin (H&E) staining for routine histological examinations.

### Statistical analysis

Statistical analysis was carried out with SPSS 17.0 software (SPSS). Data are presented as the mean  ± S.D. and comparison of two sample means was analysed by Student's *t* tests. *P*<0.05 was considered statistically significant.

## RESULTS

### Cell morphology

**Figure 1:**
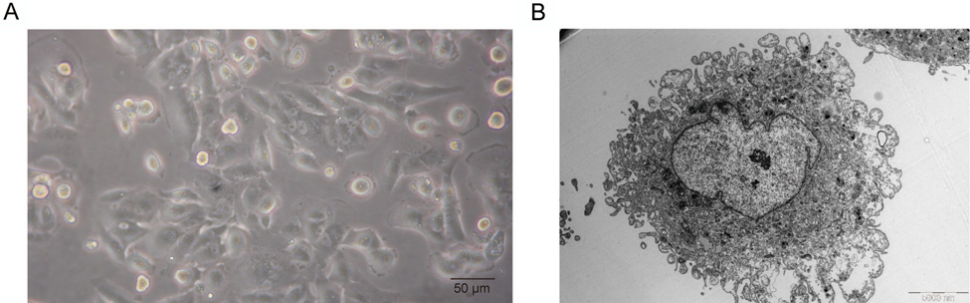
Morphologic features of THY28 cells (**A**) Phase-contrast microscopy of culture of THY28 showing clusters of polygonal cells (×40). (**B**) Electron micrograph of THY28 cell. The scale bar is 5000 nm.

THY cells adhered firmly to the culture dish. Polygonal cells with large nucleus, relatively less cytoplasm, 1–2 nucleoli and significant atypia were observed (Figure 1A). *In vitro* cultured cells were observed under an electron microscope and that showed heterogeneity with large nucleus, prominent nucleoli and dispersed chromatin, which slightly gathered in the nuclear membrane. Wrinkle-shaped depression was found on the nuclear membrane. There was less cytoplasm with lysosomes and different shapes of swollen mitochondria. Rough endoplasmic reticulum containing different swelled cisternae and free ribosomes were observed in cytoplasm (Figure 1B).

### Karyotype analysis

**Figure 2:**
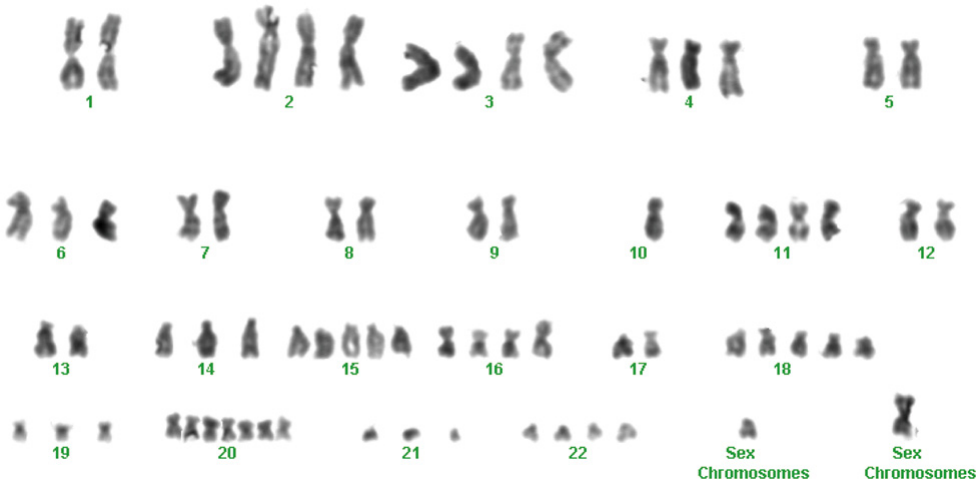
Karyotypic analysis of THY28 cells The karyotype analysis showed that THY28 cells had a human karyotype and a hypertriploid karyotype with additional numerical gains in chromosomes.

Chromosome analyses of Giemsa-banded metaphase THY28 cells revealed that the THY28 cells maintained the characteristics of chromosomes in human tumours, which were shown as polyploid cells with varied number of chromosomes mainly from 67 to 85 (Figure 2).

### Cellular growth and motility capacity

**Figure 3:**
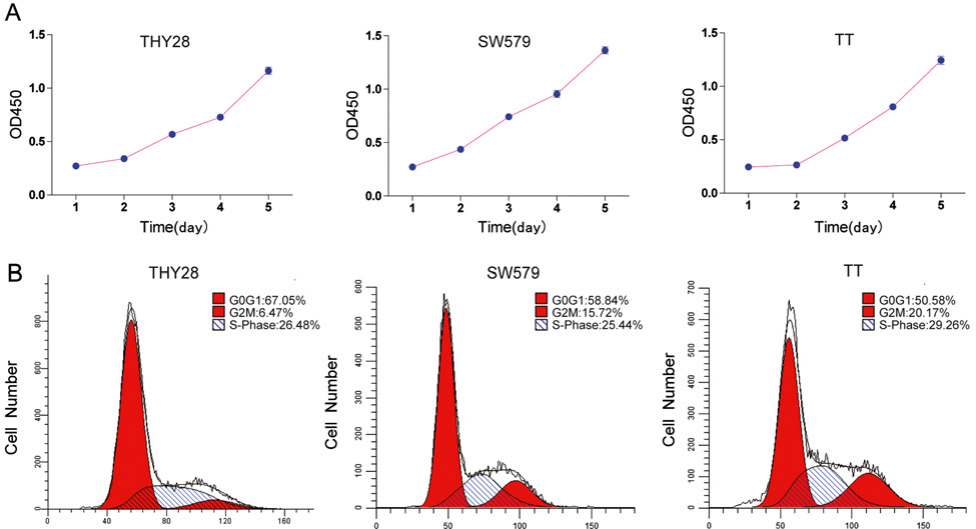
Growth rate and cell cycle of THY28 cells (**A**) Growth curve of THY28, SW579 and TT cells. Three independent experiments were performed. (**B**) Cell cycle analysis of THY28, SW579 and TT cells.

**Figure 4:**
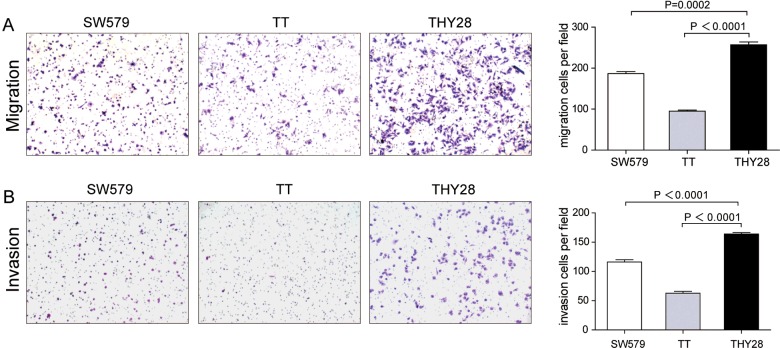
Motility of THY28 cells (**A**) Migration assays of SW579, TT and THY28 cells. (**B**) Invasion assays of SW579, TT and THY28 cells. Data represent the mean ± S.E.M.

Figure 3(A) portrays the growth curve of THY28, SW579 and TT cells. The cell concentration was increased rapidly with an average doubling time of 56 h. Results of flow cytometry assay showed that percentage of THY28 cells in G_1_-phase was 67.05% and 6.47% in G_2_-phase respectively (Figure 3B). G_1_/G_2_ was 10.36. The migration and invasion capacity of THY28 cells *in vitro* was significantly stronger than that of SW579 and TT cells (Figures 4A and 4B).

### Genes’ expression of THY28 cells

**Figure 5:**
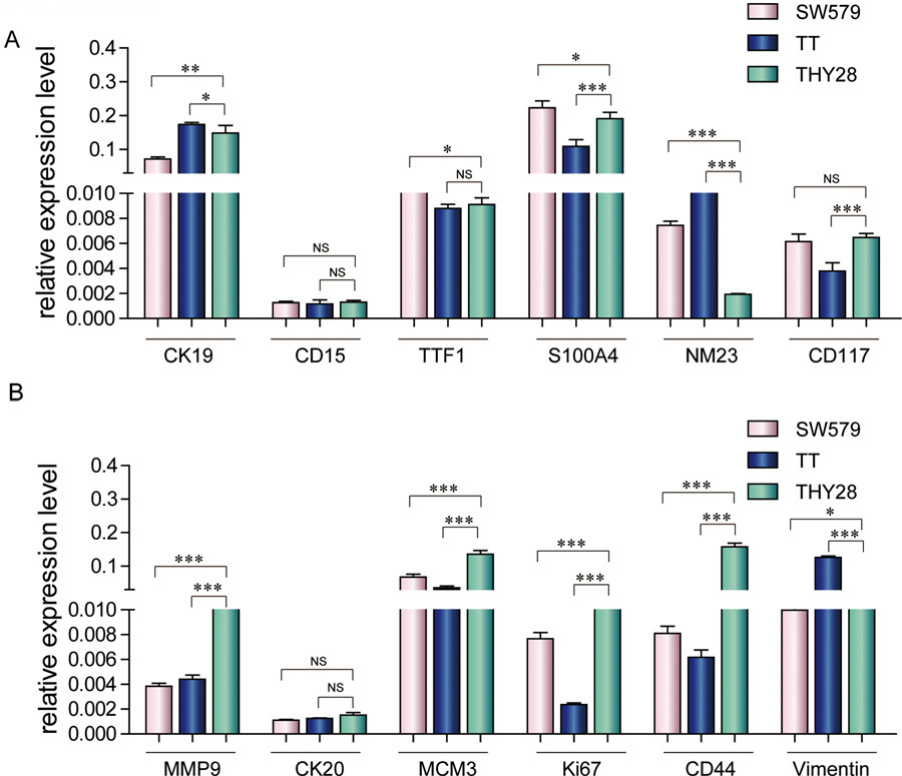
Genes expression of THY28 cells (**A** and **B**) The expression of a series of genes was analysed by real-time PCR [(A): *CK19*, *CD15*, *TTF1*, *S100A4*, *NM23* and *CD117*. (B): *MMP9*, *CK20*, *MCM3*, *Ki67*, *CD44* and *Vimentin*]; ***P<0.001, **P<0.01, *P<0.05; NS: no significance.

We analysed the expression levels of a series of genes (*CK19*, *CD15*, *TTF1*, *S100A4*, *NM23*, *CD117*, *MMP9*, *CK20*, *MCM3*, *Ki67*, *CD44 and Vimentin*) using qRT-PCR. The expression levels of *CK19*, *CD15, CK20* and *TTF1* were similar in THY28, SW579 and TT (Figures 5A and 5B). However, compared with SW579 and TT cells, different expression levels of *NM23, CD117, MMP9, Ki67* and *CD44* were shown in THY28 cells (Figures 5A and 5B).

### Cell surface biomarkers of THY28

**Figure 6:**
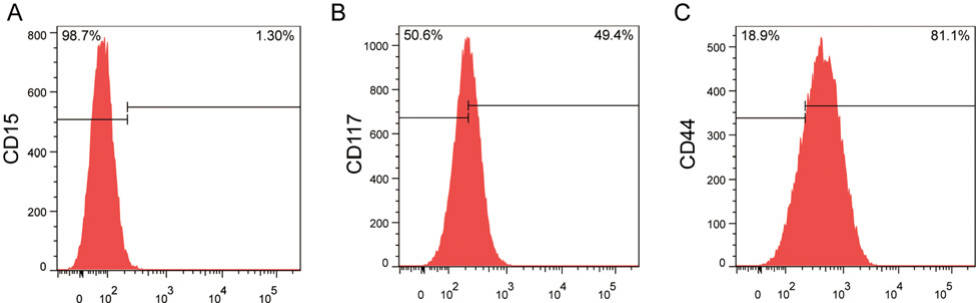
Cell Surface Markers of THY28 cells (**A**–**C**) The levels of *CD15* (A), *CD117* (B) and *CD44* (C) in THY28 cells were analysed by flow cytometry.

We analysed the expressions of *CD15*, *CD117* and *CD44* in THY28 cells by flow cytometry. *CD44* was highly expressed (81.1%) in THY28 cells (Figure 6C). THY28 cells showed a low expression of *CD15* (1.3%) and a moderate expression of *CD117* (49.4%) (Figures 6A and 6B).

### Tumorigenicity of THY28 cells and histopathological features of xenograft

**Figure 7:**
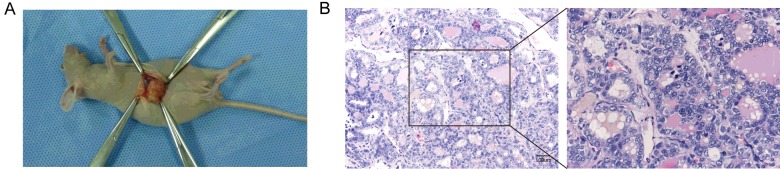
Tumorigenicity of THY28 cells in mice (**A**) Representative image of subcutaneous tumour formed in mice 6 weeks after injected subcutaneously into the flank of mice. (**B**) Histological features of paraffin embedded tissue section from the xenograft formed by THY28 cells *in vivo*. H&E staining (Scale bar: 100 μm).

**Figure 8:**
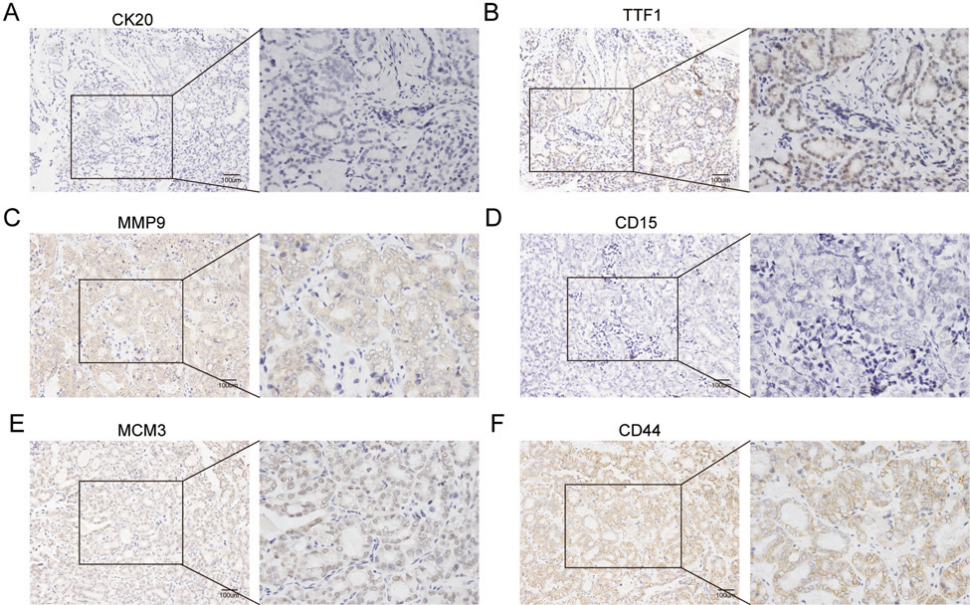
Histopathological characteristics of xenograft (**A**–**F**) By immunohistochemistry, xenograft originated from THY28 cells showed positive reactivity for (B) *TTF1*, (C) *MMP9*, (E) *MCM3* and (F) *CD44*. The expressions of (A) *CK20* and (D) *CD15* were negative in xenograft sections. The scale bar is 100 μm.

THY28 cells proliferated rapidly in immunodeficient mice *in vivo*. Visible subcutaneous tumours could be observed in 9 mice (9/10) at 6 weeks after subcutaneous injection of THY28 cells (Figure 7A). H&E staining of xenograft sections showed typical features of thyroid cancer (Figure 7B). Immunohistochemical staining showed that expression of *CD15* was negative in xenograft sections, whereas *CD44* was strongly positive in sections. In addition, *TTF1* and *MCM3* were positive in the nucleus and *MMP9* were positive in the cytoplasm (Figure 8).

## DISCUSSION

In the present study, we report the characterization of an Asian metastatic thyroid cancer cell line (THY28) from a fresh vertebrectomy specimen. This cell line was derived from a patient with the clinical and histopathological criteria of thyroid papillary carcinoma. THY28 cells were typical epithelioid cells that grew rapidly with a doubling time of 56 h. Conventional cytogenetic analyses of THY28 cells demonstrated a reproducible and hypertriploid karyotype pattern. Investigation of migration capacities between THY28 cells and SW579, TT cells showed that migration capacity of the THY28 cells was stronger than that of the SW579 and TT cells. Animal experiments indicated that transplanted tumours maintained the histopathological characteristics of thyroid carcinoma. Taken together, these results demonstrated that THY28 cells could be a useful *in vitro* cell model for studying spinal metastasis of thyroid carcinoma.

Progress has been slow in defining predictive surface markers of prognosis or clinical outcome of thyroid cancer. Previous study [18] showed that *CD117* expressions were found in most of normal thyroid tissues and half of benign thyroid lesions, but no expressions in 95% of papillary carcinomas. Murakawa et al. [19] obtained similar results that there were no *CD117* expressions in papillary carcinomas, whereas *CD117* was expressed in 40% of undifferentiated thyroid carcinomas. However, high *CD117* immunoreactivity was seen in follicular and papillary carcinomas [20]. *CD44* was expressed in prospectively identified thyroid tumour-initiating cells that induce tumours when injected orthotopically into mouse thyroid [21]. Overexpression of *CD44* in thyroid carcinoma is associated with the oncogenic conversion of the ERK signalling pathway [22,23]. Strong staining of *CD44* was found in thyroid malignancies, but both weak and negative staining were found in the tissue from the benign controls [24]. We found *CD117* and *CD44* expressions in THY28 cells were 49.4% and 81.1% respectively, but only 1.3% THY28 cells expressed in *CD15*. The presence or absence of specific marker(s) for metastatic thyroid carcinoma in Asian population requires further detailed studies for new diagnostic and therapeutic approaches.

We used a panel of genes to investigate the nature of THY28 cells. *MMP9* levels are functionally related to the metastasis and invasion properties of cancer cells. Compared with the two other thyroid cancer cell lines (SW579 and TT), higher MMP9 protein and mRNA expression were observed in THY28 cells. This high MMP9 level of THY28 cell line could be related to their metastasis to spine from a primary localized tumour. Previous research works indicated Ki-67 could be used to assess the potential risk of thyroid carcinoma metastasis [25,26], and Ki-67 was one of the most reliable indicators for detection of proliferation activity of tumour cells. The expression of *Ki67* in THY28 cells was higher than that in SW579 and TT cells, which suggested that THY28 represents the powerful proliferation and migration activity.

*NM23* is a metastasis-suppressor gene that has been proposed to play an important role in tumour metastasis suppression [27–29]. However, the higher expression of *NM23* has been associated with worse prognosis in some tumours [30,31]. Previous studies examined *NM23* expression in thyroid cancers, and the results are controversial [32–35]. Although *NM23* gene products might not be a good predictive marker for lymph node metastasis in thyroid carcinoma [36,37], in our study, *NM23* were found to be expressed at lower levels in THY28 cells compared with those in SW579 and TT cells. Interestingly, Zou et al. [37] revealed that the association of high level *NM23* expression with thyroid carcinoma suggested its correlation with rapid cell proliferation, which is not similar to our findings. Although low expression level of *NM23* was observed in THY28 cells, proliferative capacity of THY28 is far stronger than that of SW579 and TT cells in our study, which meant that THY28 cells with low *NM23* expression also had strong proliferative capacity. More studies are needed to investigate relationship between *NM23* and proliferation.

In the present study, a spinal metastatic thyroid carcinoma cell line named THY28 was established by primary culture and its biological characteristics were preliminarily studied, which laid the initial foundation for further researches on molecular mechanisms of human metastatic thyroid carcinoma. THY28 cells will provide a valuable tool through which we would understand the molecular and cellular mechanisms of metastasis in human thyroid carcinoma.

## Abbreviations

7-AAD: 7-aminoactinomycin D
CCK8: cell counting kit 8
CD15: cluster of differentiation antigen 15
CD44: cluster of differentiation antigen 44
CK20: Cytokeratin-20
DMEM: dulbecco's modified eagle's medium
H&E: haematoxylin and eosin
MCM3: minichromosome maintenance complex component 3
MMP9: matrix metallopeptidase 9
PI: propidium iodide
qRT-PCR: quantitative real-time PCR
SYBR: synergy brands
TTF1: transcription termination factor 1
